# Association of hyperuricemia with coronary heart disease: Protocol for an updated systematic review and dose-response meta-analysis

**DOI:** 10.1371/journal.pone.0308719

**Published:** 2024-08-22

**Authors:** Jinling Chen, Yi Pan, Qun Gao, Rui Zhuang, Liyong Ma

**Affiliations:** 1 Department of Cardiology, Beijing Pinggu Hospital, Beijing, China; 2 Department of Cardiology, Beijing Dongzhimen Hospital Affiliated to Beijing University of Chinese Medicine, Beijing, China; El Bosque University Faculty of Medicine: Universidad El Bosque Facultad de Medicina, COLOMBIA

## Abstract

**Introduction:**

Hyperuricemia, characterized by elevated serum uric acid levels, has garnered significant attention in cardiovascular research due to its potential association with coronary heart disease (CHD). While some studies suggest hyperuricemia as a risk factor of CHD, others present conflicting findings. A systematic review and dose-response meta-analysis is warranted to comprehensively summarize the previous studies and determine the association between hyperuricemia and CHD, thereby supporting clinical practice and future studies in this field.

**Methods:**

In this study, we will comprehensively search Medline, EMBase, Cochrane Central, ICTRP, and *ClinicalTrials*.*gov*, from inception to December 31, 2024. Prospective or retrospective cohort studies and case-control studies investigating the association between hyperuricemia and CHD will be included. Two independent reviewers will conduct study selection, data extraction, and risk of bias assessment. The primary outcome will be the pooled relative risk of CHD associated with hyperuricemia by using random-effect model. Dose-response meta-analysis will be performed with linear and non-linear model to explore the the magnitude and direction of the association between serum uric acid levels and CHD risk. Subgroup analyses will be conducted based on uric acid test approaches and corresponding cut-off values and human races. Sensitivity analyses will assess the robustness of the results with leave-one-out method, while publication bias will be evaluated using funnel plots, Egger’s test, and Begg’s test. We will further use GRADE to evaluate the quality of the evidences provided by our systematic review.

**Expected results:**

From this systematic review and dose-response meta-analysis, we hope out findings will provide reliable conclusion and data support on the association between hyperuricemia and CHD. The transparent and replicable methodologies outlined in this protocol contribute to advancing understanding of hyperuricemia as a potentially modifiable risk factor for CHD, thus supporting evidence-based strategies for cardiovascular disease management.

**Conclusions:**

This protocol describes a rigorous plan to systematically review and analyze the quantitative association between hyperuricemia and CHD risk. In a word, we will help further clinical practice and scientific studies in this field.

**Trial registration:**

This protocol was registered in PROSPERO CRD42024538553.

## Introduction

Coronary heart disease (CHD) remains a leading cause of morbidity and mortality since 1923, representing a significant public health concern [1, 2]. The adjusted death rate of cardiovascular disease is still very high as 224.4 per 100 000 people, most of which are CHD [1]. CHD encompasses a spectrum of conditions, including myocardial infarction, angina pectoris, and coronary artery disease, all characterized by the obstruction or narrowing of the coronary arteries, typically due to atherosclerosis [3].

The etiology of CHD is complex, with numerous risk factors contributing to its development and progression [4]. Traditional risk factors such as hypertension, dyslipidemia, smoking, and diabetes mellitus have been extensively studied and established in the literature [5]. However, emerging evidence suggests that hyperuricemia, defined as elevated serum uric acid levels, may also play a role in the pathogenesis of CHD [6].

Hyperuricemia has been associated with various metabolic disorders, including obesity, insulin resistance, and metabolic syndrome [7], all of which are known risk factors for CHD. Moreover, uric acid has been implicated in the inflammatory process, endothelial dysfunction, and oxidative stress [6], mechanisms that are central to the development and progression of atherosclerosis, the underlying pathology of CHD.

There have been a few studies investigated whether hyperuricemia is a risk factor of CHD, and several systematic reviews have summarized the existed evidences [8–10]. Since the first systematic review reported an increased cardiovascular mortality associated with gout in 2015 [8] and supported by following reviews [9, 10]. However, the conclusion seems to become inconsistent in 2018, the relationship between hyperuricemia and coronary heart disease remains controversial [11, 12]. This may due to the lack of enough trials in this field and the significant heterogeneity among them [11]. In addition, the included observational studies may not reflect the true effect for the presence of confounding factors and bias [11]. In the recent years, new findings from studies of relatively large sample sizes have been reported, suggest potential association between hyperuricemia and the development and progression of CHD [12–15]. This may help us reach a more reliable comclusion by including more trials. Therefore, an updated systematic review is warranted to comprehensively evaluate the existing studies, and explore the clinical implications for CHD prevention and management.

In this systematic review, we aim to synthesize the current evidence from cohort studies and case-control studies of participants without hyperuricemia and CHD at baseline and reported the occurrence of hyperuricemia and CHD in the follow-up period, to regard the relationship between hyperuricemia and CHD, critically evaluate the strengths and limitations of existing studies, and identify key gaps in knowledge that warrant further investigation. By enhancing our understanding of the interplay between hyperuricemia and CHD, we can potentially identify novel therapeutic targets and interventions to mitigate the burden of this prevalent cardiovascular disease.

## Material and methods

This protocol for a systematic review and dose-response meta-analysis follows the Preferred Reporting Items for Systematic Reviews and Meta-analyses (PRISMA) protocol (PRISMA-P) statement (S1 Checklist) [16]. The systematic review and dose-response meta-analysis will be conducted following the Cochrane Handbook [17] and Meta-analysis Of Observational Studies in Epidemiology (MOOSE) guidelines [18], reported following the PRISMA statement [19]. This protocol was registered with PROSPERO (CRD42024538553).

### Search strategy

We will search the Medline, EMBase, and Cochrane CENTRAL from inception to December 31, 2024, to identify publications investigated the association between hyperuricemia and CHD. The International Clinical Trials Registry Platform (ICTRP) and the *ClinicalTrials*.*gov* will also be searched for unpublished data. We will also check the reference lists of the reviews in this field and the potentially included articles to avoid missing studies. We will include publications in all languages. We will also contact the corresponding authors of the included articles after full text screening for more grey literature or grey data. The search strategy for Medline-PubMed is presented as Table 1 as an example.

**Table 1:** Search strategy for medline (PubMed).

Index	Search Terms
#1	“Uric acid”[Mesh]
#2	“uric acid”[title/abstract] OR “urate” [title/abstract] OR “hyperuricemia” [title/abstract]
#3	“Coronary disease”[Mesh]
#4	“coronary heart disease”[title/abstract] OR “coronary artery disease”[title/abstract] OR "ischemic heart disease"[title/abstract] OR “myocardial infarction”[title/abstract] OR “angina pectoris”[title/abstract] OR “unstable angina” [title/abstract]
#5	“Cohort studies”[Mesh]
#6	“cohort study” OR “longitudinal study” OR “prospective study” OR “follow-up study” OR “case control” OR “case-control” OR “case-comparison study” OR "retrospective study"
#7	#1 OR #2
#8	#3 OR #4
#9	#5 OR #6
#10	#7 OR #8 OR #9

### Eligibility criteria

We will identify articles for this systematic review and meta-analysis following the criteria as below:

1. Patients: We will only include studies that enrolled human adults with no cardiovascular disease or gout at baseline, and diagnosed with hyperuricemia in the follow-up period in this systematic review. We will set no limitation on the demographic indicators including sex, human race, region, comorbidities, etc.
2. Exposure: The exposure is the occurrence of hyperuricemia among the participants during the follow-up period from original studies. The included studies should report the exact value of serum uric acid and the approach to test the serum uric acid in the text, and provided the cut-off value and diagnostic criteria for hyperuricemia.
3. Control: The participants regarded as the control group are those never diagnosed with hyperuricemia at neither baseline nor the follow-up period in the original studies.
4. Outcome: The outcome of this systematic review will be the difference between the occurrence proportions of CHD among patient group and control group, to test whether hyperuricemia is a risk factor for CHD or not.
5. Study design: We will only include prospective or retrospective cohort studies or case-control studies in this systematic review. The included studies should have at least 100 participants of sample size, with at least 1 year of follow-up period.

### Study selection

**Fig 1:**
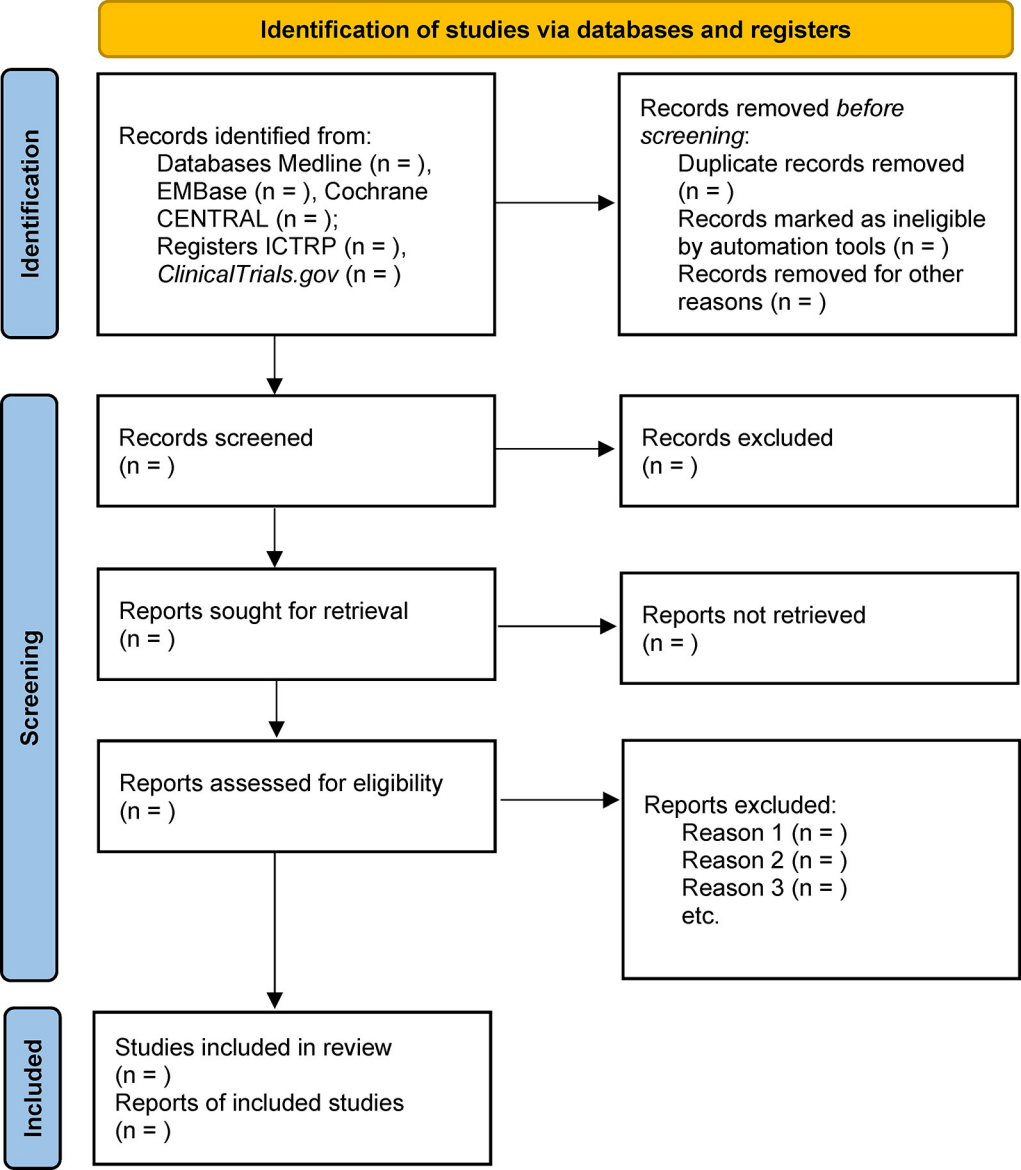
Flow diagram of the systematic review.

Two reviewers (JC and RZ) will screen the retrieved records independently after remove duplications by using EndNote 20 software. The first step will screen the titles and abstracts to remove most of the unrelated records. After that, the two reviewers will go through the full text of each record to select those that meet the eligible criteria. Any disagreement will be solved by a third reviewer (LM), and further discussed within all reviewers if not solved. The whole process of study selection will be presented as Fig 1.

### Data extraction

Two independent reviewers (JL and YP) will extract the required information and study data with Microsoft Excel software individually. The required information includes (1) publication information: name of the authors, publication year, country or region, etc.; (2) demographic data: human race, age, sex, BMI, comorbidities and drugs, etc.; (3) study data: the test approach and exact values of serum uric acid, the time and cut-off value for the diagnosis of hyperuricemia, the diagnosis criteria of CHD, etc.; (4) other information: attendance rate, reasons for withdrawing, etc. The publication information, demographic data, and other information will be recorded as text, exact number with proportion, or mean±standard deviation (SD). Study data will be recorded as hazard ratio (HR) with 95% confidence interval (CI) from the original studies, however, odds ratio (OR), risk ratio (RR), or the exact proportion of participants with CHD in each cohort or case series after follow-up will also be recorded. The above data will be verified by comparing between the two reviewers, and any disagreement will be carefully checked by the third reviewer (LM). Missing information and missing data will be obtained by sending e-mail to the corresponding authors when necessary.

### Quality assessment

We will assess the methodological quality of the included studies with the tool to assess risk of bias in non-randomized follow-up studies of exposure effects (ROBINS-E) [20]. The ROBINS-E tool provides a reliable approach to evaluate the risk of bias of observational studies. The assessment addresses bias within seven domains, through a series of ‘signalling questions’ with options ‘Yes’, ‘Probably yes’, ‘Probably no’, ‘No’ and ‘No information’. The seven domains include bias due to confounding, bias arising from measurement of the exposure, bias in selection of participants into the study (or into the analysis), bias due to post-exposure interventions, bias due to missing data, bias arising from measurement of the outcome, and bias in selection of the reported result. An overall bias should be assessed with the assessment of the seven domains of risk of bias. For missing information, we will contact the corresponding authors by sending e-mail and search the registration databases.

### Qualitative and quantitative synthesis

#### Qualitative synthesis

For the qualitative synthesis, we will present the summary of the included studies as a table to report the characteristics and necessary information. The first author and publication year, key demographic information (human race, age, sex, etc.), study design (including sample size and follow-up time), and diagnose criteria for hyperuricemia and CHD will be summarized in the table. After that, we will summarize the study design and result of each individual study in text to present a general summary of the systematic review.

#### Quantitative synthesis

We will conduct a pooled estimation for each comparison with HR values. The ORs will be transferred to RRs when necessary [21], and RRs will be regarded as HRs for the meta-analyses. The random-effect model will be used for all comparisons, while the *I*^*2*^ value and τ^2^ will also be calculated and reported to assess the potential heterogeneity among included studies. We will present forest plots with pooled estimations for those with *I*^*2*^ value less than 90%, and forest plots without pooled estimations for those *I*^*2*^ value more than 90%. We will also perform subgroup analysis and dose-response meta-analysis to further test the potential association between hyperuricemia and CHD. The R software v4.3.1 [22] with metafor package [23] will be used for quantitative synthesis.

### Subgroup analysis

We will further analyze and explain the results with subgroup analysis. The pooled estimations will be divided into subgroups by prospective or retrospective cohorts, different test approaches and corresponding cut-off values of uric acid, different outcome measurements (RR, OR, or HR), and demographic factors including human races, sex, dietary habits, alcohol consumption, nutritional status, comorbidities, metabolic risk factors, and medications if possible, to test the potential confounding factors for the association between hyperuricemia and CHD.

### Sensitivity analysis

After subgroup analysis, we will perform a sensitivity analysis by excluding studies one by one with the R command ‘leave1out’ from metafor package, to observe the effect of a single study on the pooled estimation. Significant changes caused by the exclusion of a single study may indicate significant heterogeneity between the single study and the others, and should be explained and discussed carefully.

### Assessment of publication bias

We will use funnel plot with Egger’s test and Begg’s test to detect potential publication bias while no less than 10 original studies are included in a pooled estimation. We will also use trim-and-fill approach to test the relative effect of publication bias on the pooled estimation when possible.

### Dose-response meta-anaylsis

If data are available, we will apply dose-response meta-analysis with R package dosresmeta and the command ‘dosresmeta’ [24] to further explore the association between uric acid and CHD. We will use the absolute values of uric acid from each original studies for the first step, and then divide the absolute values by the cut-off value for the diagnosis of hyperuricemia of each original studies for the second step, to verify whether the test approaches and corresponding cut-off values should be considered in clinical application. Both linear trend and non-linear trend will be tested and reported in our meta-analysis. Potential confounding factors will be regressed out in the analysis, including those mentioned in the subgroup analysis.

### Evidence grade evaluation

We will use GRADEpro V3.6.0 software to assess the quality of each outcome’s evidence grade under the guidance of the Grading of Recommendations Assessment, Development and Evaluation (GRADE) recommendation [25]. Since this systematic review will only include cohort studies and case-control studies but not randomized controlled trials, the following items can be the reasons to decrease quality of evidence: limitations in study design, inconsistency, indirectness, imprecision, and publication bias. The following items can be the reasons to increase quality of evidence: large effect, plausible confounding would change the effect, dose-response relationship. We will provide a “Summary of Findings” table to summarize the quality of the evidences in the systematic review.

### Ethics and dissemination

This study does not require formal ethical approval. The findings will be submitted for publication in a peer-review journal.

### Patients and public involvement

As this is a protocol for our systematic review, we will obtain public data from published literatures or from the authors of the original studies. Thus, patients or the public will not be involved in the design, or conduct, or reporting, or dissemination plans of our research.

## Discussion

This systematic review and dose-response meta-analysis will comprehensively summarize and analyze the existing results of previous cohort studies and case-control studies to determine the association between hyperuricemia and CHD.

Metabolic disorders such as hyperlipidaemia, diabetes, and hyperhomocysteinaemia, are recognised risk factors for CHD [26]. Controlling blood glucose, homocysteine, and lipids, especially low-density lipoprotein cholesterol, are effective treatments for CHD and have been proved to significantly improve long-term prognosis [27]. Anti-metabolic disorder drugs have become reliable treatments for CHD. Drugs, such as sodium-dependent glucose transporters 2 inhibitors (SGLT2i) and glucagon-like peptide-1 receptor agonist (GLP-1RA), have further been demonstrated to have cardiovascular protective effects independent of glucose regulation [27]. Hyperuricemia is also a metabolic disease, but its association with CHD is not yet clear [12]. Previous systematic reviews in this field have reported controversial conclusions on the association between hyperuricemia and CHD, made it hard to decide whether serum uric acid should be controlled in population with high risk of CHD and population with CHD. It remains unknown of the best range of serum uric acid to be controlled among the population as well [28]. Since several articles reported new findings from the latest studies, it is necessary to update the systematic review and meta-analysis to investigate the association between hyperuricemia and CHD, and further perform dose-response meta-analysis to explore the quantitative association.

There are some limitations about this systematic review. We will only search databases and clinical trials registration platforms in English. We may miss some valuable studies published in other languages. In addition, although our systematic review will include only participants without CHD and gout at baseline, the participants may already have arteriosclerosis or have had undiagnosed hyperuricemia. This may affect the reliability of the pooled estimations and the conclusion of this systematic review.

From this systematic review and dose-response meta-analysis, we hope out findings will provide reliable conclusion and data support on the association between hyperuricemia and CHD. In a word, we will help further clinical practice and scientific studies in this field.

## Supporting information

S1 Checklist PRISMA-P (Preferred Reporting Items for Systematic review and Meta-Analysis Protocols) 2015 checklist. (PDF)
